# Traversing the Funambulist’s Fine Line between Nursing and Male Identity: A Systematic Review of the Factors that Influence Men as They Seek to Navigate the Nursing Profession

**DOI:** 10.3390/ejihpe10030051

**Published:** 2020-07-05

**Authors:** Daniel Terry, Blake Peck, Clarissa Carden, Alicia J. Perkins, Andrew Smith

**Affiliations:** 1 School of Health, Federation University, 3350 Ballarat, Australia; b.peck@federation.edu.au (B.P.); a.perkins@federation.edu.au (A.J.P.); andrew.smith@federation.edu.au (A.S.); 2 Griffith Centre for Social Cultural Research, Griffith University, 4111 Nathan, Australia; clarissa.carden@griffith.edu.au

**Keywords:** nurses, men, male, stereotype, workforce, recruitment, retention

## Abstract

Nursing has seen a dominance of women within the profession, and today, the presence of men in the role remains less understood and appreciated. Males considering or entering nursing face challenges concerning role misconception, marginalization, and gender bias. With a looming shortage of nurses on the horizon, it is more important now than ever before to find better ways of engaging males into nursing. The aim of the study was to examine the psychological constructs that influence male perceptions of nursing as they seek to navigate the profession, and what aspects influence men to consider nursing as a career. To achieve this, a systematic review and mixed research synthesis (integrated design) was conducted. English language research published between 1999 and 2019 was eligible. The methodological rigor of qualitative articles followed the Critical Appraisal Skills Program, while the Best Evidence Medical Education guided the quantitative review. Among the 24 publications identified, three sub-themes emerged from the overarching theme of the funambulist or tightrope walker. Sub-themes included societal, inner and collective voices that inform men’s place in nursing or their decision making about entering the profession. There is a need to re-visit what it means to be a nurse in order to address the gendered stereotypes that impact men entering the nursing profession.

## 1. Introduction

While nursing is plagued with workforce shortages across the globe [1], nurses still represent the single largest category of healthcare worker across the globe [2]. A key strategy to address this impending workforce shortage is to improve the recruitment of males into nursing [3,4]. It is however questionable whether this strategy will yield success given the longstanding historical and socio-cultural evidence in support of fewer men enrolling in programs of nursing study [5].

It is now a widely held view in contemporary thought that gender alone is not grounds for acceptance or preference into a professional body of knowledge and subsequent role. Successful examples of societies efforts to improve gender diversity can be seen in areas such as the sciences, engineering, medicine, and corporate roles, which have traditionally been over-represented by males [6]. Despite this, there remains a marked gender imbalance in nursing [7]. In Australia males make up a mere 11% of the nursing workforce [8], a picture seen time and again in other parts of the word: 9% in New Zealand, 9.6% in the United States, and 11% in the United Kingdom [5]. Scholars suggest a number of reasons to explain these low global rates of males entering nursing that tend to focus on the role of negative stereotypes [5,9,10], and a pervasive societal construction of nursing being squarely aligned with women’s work [5,7,11].

A common thread across much of the research in the area of men in nursing is the role and influence of Florence Nightingale. Considered a pioneer of modern nursing, she worked to develop training institutions that were exclusively for women [7,12]. Nightingale believed that nursing was an unsuitable role for men—a belief that has arguably played a significant role in the invisibility of men in nursing and contributed to the socio-cultural norms that influence modern culture [5,12]. 

What is evident is that the forces that shape the role and scope of males in the profession of nursing are most likely multi-factorial in nature [5]. There can be little argument therefore that an interplay of some deep-seated sociocultural factors that influence males’ decisions to come into nursing as a career exists and needs to be explored if we are to better target or open up nursing to men in our societies. This review of the literature therefore seeks to distil the findings from previous studies that also seek to shed light on male constructions of nursing. Importantly, we seek to understand both the perspective of those males engaged in the nursing profession to better understand the insider perspective and the perspective of those males not engaged with nursing, so as to understand better the sociocultural perceptions of nursing that pervade our societies and influence our men.

### Research Aim

The aim of the systematic mixed research synthesis is to (i) systematically examine the psychological constructs that influence male perceptions of nursing as they seek to work in and navigate the profession, and (ii) to identify aspects such as attraction and barriers that determine a male considering nursing as a career.

## 2. Materials and Methods 

A systematic review and mixed research synthesis—an integrated design informed by the work of Sandelowski et al. [13]—was utilized as a framework to navigate the integration of results from both qualitative and quantitative studies within this review. Mixed research synthesis recognizes the contribution that diverse forms of evidence have in evidence-based practice and that informs many disciplines [13]. The underutilization of qualitative studies has been amplified by the requirement of applied evidence-based practice modalities to be informed by empirical evidence in the traditional sense, situating qualitative studies as unscientific and unworthy in the hierarchy of evidence in which randomized clinical trials and quantitative meta-analyses are highest. Within this debate there are those who tolerate the contribution of qualitative research to evidence-based practice by default rather than by design, on the one hand, and those who argue that qualitative research is a necessity in achieving the goals of evidence-based practice [14]. There are facets of human experience that are not reached by the gold-standard of randomized controlled trials and yet at the same time are central in developing participant-centred practical significance. 

Recognizing that for each researcher the inherent differences between qualitative and quantitative research have an influence upon the research design, Sandelowski et al. [15] proposed three designs—segregated, integrated, and contingent—that accommodate different perspectives on the relationship between qualitative and quantitative research. For the purpose of the present study, an integrated approach was used.

An integrated approach is based upon a series of assumptions that culminate in a view that the theoretical differences between qualitative and quantitative are not insurmountable. Here researchers grouped together targeted areas of interest not on the basis of their methodological approach, but rather by the findings that are viewed as answering the very same question and are therefore convertible [14]. Unlike a segregated approach that requires the researcher to adhere to the quantitative and qualitative binary and, therefore, to group findings on the basis of methodology, in an integrated perspective findings are grouped on the basis of thematic similarities in the findings [13].

The systematic review and integrated mixed research synthesis as guided by Sandelowski, Voils, and Barroso [15] was used to identify, evaluate, and synthesize quantitative and qualitative research-based evidence in order to answer the research questions. The objectives, analysis methods and inclusion and exclusion criteria were developed and documented to ensure accurate and complete reporting of findings [13,14], following the guidelines outlined by Preferred Reporting Items for Systematic Reviews and Meta-Analyses (PRISMA) [16] to ensure accurate and complete reporting of findings.

### 2.1. Search Strategy

A search of the literature was run on ProQuest Central, Web of Science Core Collection, Scopus, Medline, and Cumulative Index of Nursing and Allied Health Literature (CINAHL) was completed on November 10, 2019 for studies related to men in nursing. The search examined title, keyword, and abstract, adapted to each database’s specific requirements, with a search strings that included: “nurs*” AND “male” OR “man” OR “men” OR “masc*” AND “stereotypes” OR “representation” OR “perceptions” OR “attitudes” OR “deci*” AND “career” OR “higher education” OR “tertiary education” OR “workforce”. An additional hand search of reference lists was conducted to identify any extra studies. 

### 2.2. Inclusion and Exclusion Criteria

Studies included were those between 1999 and 2019 that were original research, including literature reviews, regardless of nursing specialty, clinical context, or level of nursing or health education. After duplicates were removed, studies were excluded if they were review articles or peer and non-peer reviewed commentaries regarding men in nursing. It must be noted that publications prior to 1999 were initially considered; however, it was agreed that the systematic review should encompass a contemporary (21st century) political and social view and lens of men in nursing, and therefore any potential publications prior to 1999 (n = 29) were also excluded. Full-text articles published in languages other than English were excluded due to issues associated with translation quality. 

### 2.3. Study Screening

Articles retrieved from the search were uploaded to Rayyan, a free application used for systematic reviews [17], and two of the authors (CC and DT) undertook a blinded review of the title, keyword, and abstract of the downloaded items after duplicates and exclusions were removed. A second round review of full text articles were then assessed independently and judged against the inclusion and exclusion criteria by two reviewers (CC and DT) with each article reviewed being classified as “include”, “exclude”, or “not sure”. Any discrepancies between reviewers were resolved by discussion with a third reviewer (BP) until consensus was achieved. 

### 2.4. Methodological Quality Assessment Procedure 

The methodological quality of the included studies was assessed independently by two reviewers (CC and DT). The scoring of the each qualitative study occurred using the Critical Appraisal Skills Program (CASP) tool for qualitative research [18]. The quality of the qualitative papers were scored as “met” (1), “partially met” (0.5), and “not met” (0) with scores added to gain a final score of 9.0–10.0 (high quality), 7.5–9.0 (moderate quality), 6.0–7.5 (low quality), or 0.0–6.0 (exclude) [18]. Eight (72.7%) out of eleven qualitative publications had a score of more than 6.0 and were considered to be of high methodological quality and included in the review, as presented in Table 1. It must be noted that all authors involved with the review and methodological assessment (BP, CC, and DT) have completed Doctor of Philosophy (PhD) research studies and post-doctoral research training, in addition to having extensive experience in and being fully conversant with undertaking systematic reviews.

In addition, the methodological assessment for quantitative studies was initially undertaken against Cochrane quality criteria as outlined by Higgin and Green [30]. However, all studies would have been excluded due to failing to meet the criteria of randomisation, assessors being blinded to outcome, or blinding of participants. Therefore, the Cochrane quality criteria were inappropriate for the studies highlighted. Although there is no one specific methodological assessment tool developed for observational studies, Sanderson et al. [31], indicated that there are a plethora of instruments with various methods of examining risk of bias. In addition, it remains inappropriate to use the Strengthening the Reporting of Observational Studies in Epidemiology (STROBE) statement, as it was not developed to be used as a tool for assessing the quality of observational research [32]. Therefore, the quality of the quantitative papers was rated against the 11 Best Evidence Medical Education (BEME) quality indicators developed by Buckley et al. [33]. These indicators were developed to ascertain the appropriateness and quality of studies, including study design, its conduct, analysis of results, and conclusions made, where higher-quality studies are those that meet the minimum of seven of the eleven indicators [33]. Using the BEME indicators and within this context, criteria are examined as either criteria being “met” (+), “not met” (−), or “not applicable” (n/a). Overall, this led to a 95% agreement between the reviewers, with any disagreements being discussed and a third reviewer (BP) being consulted to reach consensus. The quality score of the quantitative publications ranged from 5 to 9, while 13 publications of the total 14 (93%) publications had a score of more than seven and were considered to be of high methodological quality and thus included in the review presented in Table 2. Lastly, the methodological quality of literature reviews was not examined, but remained included within the accepted studies. 

### 2.5. Data Extraction and Analysis

Informed by the approach to qualitative systematic review outlined by Sandelowski, Voils, and Barroso [15], the data extraction was undertaken by reviewers (CC and DT) who extracted all data using Microsoft Word. Following a modified version of the process outlined by Colaizzi [48], each reviewer (CC, DT, and BP) independently read and re-read each article identified in order to formulate significant statements and meaning, as well as the interpretation, ideas, accounts, and assumptions of what the findings presented by the authors of each of the identified papers represented. Reviewers then shared their interpretation of the articles resulting from the independent review. Here common or recurring patterns in the significant statements and meanings amongst the significant statements and understandings identified from the independent review process were aggregated and formulated into thematic representations to describe the phenomena. 

The quantitative approach for mixed research synthesis was informed by Voils et al. [49] and Crandell et al. [50]; however, the vast heterogeneity of research articles’ hypotheses, research questions, and findings of each individual study precluded undertaking in-depth meta-analysis. Data included continuous data, categorical data, or themes, and in some cases data were not accurately reported, leaving gaps within elements of the data. For example, articles would report a significant finding; however, they would leave out key statistical information [34]. Therefore, the quantitative data analysis was modified to address these deficits, while it remained informed by Sandelowski, Voils, Crandell, and Leeman [13], and Sandelowski, Voils, and Barroso [15]. 

To address this, when data were extracted from the quantitative studies, results, outcomes, and findings were grouped into ten topics or domains, which were then truncated with other similar topics due to their parallelism or relevance to other overlapping topics or domains leaving three overarching domains to undertake data analysis. Due the diversity and quality of the data extracted, only descriptive statistics of the data and key findings from each study were analysed. Again, each reviewer (DT and BP) independently examined each article in order to identify and articulate significant findings and meaning from the qualitative data, while developing an interpretation of what the collective quantitative data were presenting from the identified papers. 

Remaining consistent with the application of the mixed research synthesis integrated design, each reviewer (CC, DT, and BP) employed the process of aggregation, where findings that had been identified as communicating the same understanding of the phenomena of interest were grouped together as a confirmation of the finding. On the other hand, the reviewers also used the process of configuration whereby findings that are thematically diverse and therefore not amendable to pooling were brought together to contradict, extend, explain, or otherwise modify each other [13]. In culmination, the findings from this process are presented below in the form of a traditional thematic analysis with areas of aggregation and of configuration identified. 

## 3. Results

**Figure 1:**
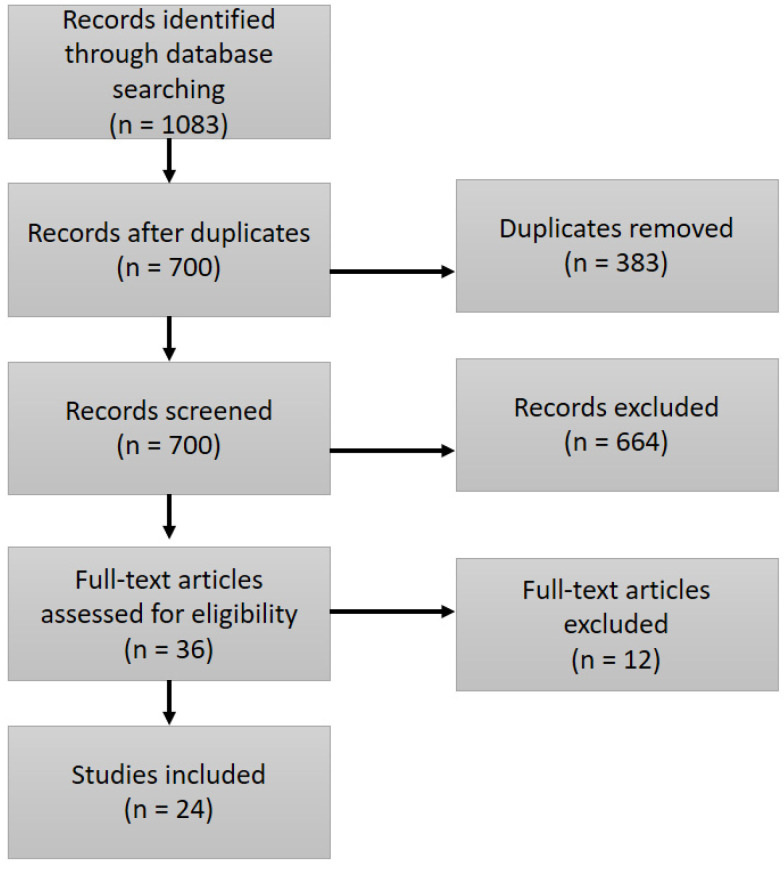
Systematic review flow chart.

The systematic search yielded 1083 potentially relevant publications and after removing duplicates (n = 383) and those that did not meet the inclusion criteria (n = 658), a total of 42 were agreed upon. A further six were excluded, as full-texts could not be obtained, leaving 36 items to be included in the full-text review. After the review of each article identified, using CASP and BEME to ensure the research quality, twelve additional articles were excluded. For example, four of these articles were brief review pieces, four had poor methodological quality, and five were poorly translated into English and could not be deemed appropriate for inclusion by the authors, as outlined in Figure 1. Overall, 24 publications were included in the final group of publications, which included nine qualitative studies, 13 quantitative studies, one literature review [51], and a concept analysis [52].

Two of the articles did not have a clear country of focus, as these were a concept analysis [52] and a literature review [51]. Of the remaining articles, the most common countries of focus were Canada (n = 6), the United States (n = 5), and Australia (n = 5), with other countries, such as Ireland, New Zealand, Israel, and Jordan also represented in separate studies. Overall, just over half (n = 13) of the studies focused on undergraduate students, while two (n = 2) additional studies focused on or included postgraduate students. In addition, half (n = 12) of the studies focused solely on male nurses, while another five (n = 5) studied both male and female nurses. Lastly, just over one third of the articles (n = 12) relied on questionnaires, while eight articles used interviews or focus groups as the data collection method, as outlined in Table 3. 

Three key sub-themes from the qualitative literature and the quantitative data were identified and emerged from the overarching theme of the funambulist or tightrope walker. The central theme highlights the competing worldviews, societal voices, and collective agendas that men must precariously comprehend, negotiate, and traverse as they seek to learn, work in, and navigate the nursing profession. The first sub-theme from the literature findings is the scrutinizing voice that questions “why are you participating?” and encompasses societal stereotypes, perceptions, and gender norms. The second sub-theme is the inner voice or monologue that questions “why am I participating?”, incorporating the rewards of nursing as career. Lastly, the third and final sub-theme is centred on the collective voice that indicates “why you should be participating”, focusing on male role models, family, and familiarity with the profession. Each of these sub-themes or voices that encompass the challenges and strategies to improve men entering nursing are discussed in detail below.

### 3.1. Why are You Participating—Societal Stereotypes, Perceptions, and Gender Norms

One of the key challenges for males entering or being an active member of the nursing profession is the perception that nursing as a profession is observed as an inferior career choice relative to other health-related professions, such as Medicine [51]. In addition, other key difficulties experienced included the stereotype that male nurses are homosexual, that it was not a “macho” type of career as nursing is feminine role, and men participating in the profession are effeminate or are undertaking female-oriented occupation, or what some might consider as “women’s work” [25,35,36,42,51]. Within the literature elsewhere, this was also observed to be a barrier, particularly among male nursing students and those seeking to enter post-secondary or higher education [19,34,41]. 

Among a number of students, these stereotypes of what constitutes a “nurse” were shown to make the feminization of the nursing curriculum and nurse education uncomfortable or less conducive for some men, and has led to a decrease in retention of male nursing students within post-secondary or higher education [35,41,51]. For example, male nursing students in a Canadian study conducted by Twomey and Meadus [47] indicated they felt that they “stood out” and this was a contributing factor that impacted their experience in both education and practical settings.

These stereotypes were further evident due to a perceived incongruence between masculinity and the nursing profession, which was a source of tension and difficulty for male nurses to navigate [25,37]. Nevertheless, a study conducted by Loughrey [39] in Ireland had found that many male nurses were able to or had the capacity to identify more with female than male gender norms. In addition, Loughrey [39] and Thompson, Glenn, and Vertein [46] demonstrated that nurses who were men had significantly lower masculinity levels, while their femininity levels were commensurate with their male non-nursing counterparts. Overall, it was indicated that both male and female nurses were significantly more androgynous than their non-nursing counterparts [46]. Although this group of males demonstrated male gender norms, it was suggested that as a group male nurses exhibited “soft” masculinity, where they maintain certain socially accepted or stereotypical male qualities, while “regarding themselves as affectionate, sympathetic and understanding” [39]. Despite this finding, men and students within the nursing profession felt there was a level of discrimination toward them, in that they were seen and relied on as the “muscle” or those who could or should handle more aggressive, violent, or difficult patients [4,39,45,47].

The soft masculinity reported amongst men in Ireland [39] can be considered to be in contrast to more negative stereotypes represented in studies from Jordan and Israel [19,34]. It is suggested that geographical areas with typically stronger religio-cultural norms tend to also have more traditional gender roles. Conversely, in a United States study it was indicated that nursing students had reported higher levels of masculinity and femininity than reported in previous USA studies, which suggests a change may be occurring within nursing regarding the relationship between gender norms and the profession [46]. Despite this, Sasa [52], has argued that the very idea of the term “male nurse”, so ubiquitously used within the profession, within the healthcare workforce, and society, including the media, continues to perpetuate gendered stereotypes about who should participate and what nursing should resemble [47]. 

### 3.2. Why am I Participating—The Rewards of Nursing as A Career 

Beyond the stereotypes, perceptions, and gender norms that appear to permeate the nursing profession for and amongst men, another theme was identified that centred on the reward that males were able to glean from being a member of the nursing profession. The key rewards that were important to males to enter or be a nurse were focused on the financial remuneration, including stable employment, job security, the level of pay, leave entitlements, and extra benefits, and were identified within several studies [4,24,34,38,45,47]. In addition to financial reward, the perceived career stability as a nurse was demonstrated as a motivating factor for men to enter the nursing profession [20,47]. However, financial factors were also found to be a major motivator for men leaving the nursing profession [45,47], with one study conducted by Rajapaksa and Rothstein [43] in the USA demonstrating that men were 2.5 times more likely to leave nursing for financial reasons than women. 

Other aspects that men found rewarding beyond the financial elements of the career were shown to be the caring aspect of nursing, which encompassed altruism and the capacity to make a difference [4,45]. However, it was suggested that men in nursing felt they needed to precariously traverse their caring roles, leaving themselves in vulnerable positions, as they often felt as though they were perceived as having deviant or sexualized motives behind their desires to provide care [4,24,51]. By contrast, it has also been identified by male nurses that they are perceived as less caring than their female nursing counterparts, and therefore not well suited to the profession, leaving males perplexed, disconcerted, and dissatisfied [4,44,45].

Regardless of the perceptions of others, caring among men was deemed personally rewarding and essential for them to be part of and stay in the nursing profession [23,26,29,47]. Among males already engaged as nurses there was a sense of meaning, purpose, fulfilment, and satisfaction from key elements of the work, such as making a difference in other people’s lives and the work itself being both challenging and rewarding, especially among men who had previously worked in other professions [23,35,45,47]. Overall, the study conducted by Rochlen, Good, and Carver [44] indicated that despite the challenges males within the nursing profession encountered, they were more satisfied, on average, than men in other professions. 

### 3.3. Why You Should be Participating—Male Role Models, Family, and Familiarity 

The last theme had identified that men were greatly influenced to consider, commence, and remain in the nursing profession by way of other male role models who are present in the profession [4,25,26]. Further, often men in nursing became familiar with the nursing profession and what nurses actually do through others or even their own previous employment around nurses and nursing, which often means males enter the profession later in life than their female counterparts [4,25,26,45]. 

Familiarity with the role of nursing is generally less well understood by the public, and therefore, males indicated their understanding and desire to enter the profession was achieved through others. For example, supportive family members, including parents, siblings, extended family, and close associates who were either nurses or working in health were vital in the decision making process [4,25,26,45,51]. In addition, familiarity with nursing was also achieved through a natural progression or an advancement among men who had worked in other health-related employment, due to their own helping experiences, or because of their interactions with nurses [24,25,26,51]. However, it was also indicated that less supportive family members, including female nursing students, can also have an impact on, or not be supportive of, male family members who seek to pursue a nursing career [35,42].

Further, familiarity with nurses and what they do is also shown to be an important factor in mitigating the impact of negative stereotypes toward nursing as a profession for men [23]. For example, in a Canadian study conducted by Clow, Ricciardelli, and Bartfay [34], it was demonstrated that greater exposure to men in nursing was associated with increased positive attitudes and stereotypes of men in this profession. However, television representations of male nurses have been shown to be implicitly or unintentionally stereotypical even when they explicitly seek to challenge negative stereotypes [28]. Therefore, despite positive role models that men may have contact with, the media may further perpetuate and reinforce certain effeminate or homosexual stereotypes, portray hyper-masculine personas as a counterpoint, give male nurses minimal screen time, or leave them absent altogether [28,35,42,51]. Unfortunately, such endeavours ultimately problematize men in the nursing profession and further impair the capacity of the profession to recruit and retain men [28,42]. 

## 4. Discussion

The purpose of this systematic mixed studies review was to (i) systematically examine the psychological constructs that influence male perceptions of nursing and (ii) to identify those aspects that determine a male considering nursing as a career. The nature of these research objectives required a clear search strategy inclusive of strict eligibility criteria inclusive of an extensive and explorative search strategy. A mixed-research synthesis, and more specifically an integrated design informed by the work of Sandelowski, Voils, and Barroso [15] and Sandelowski, Voils, Crandell, and Leeman [13] led to nine qualitative studies and 13 quantitative studies being included. The results of the review yielded three areas of commonality that centred on the overarching theme of the funambulist, which highlights the competing world views, societal voices, and collective agendas that men must precariously traverse prior to and when in the nursing profession. The sub-themes are societal, inner, and collective voices that ask “why are you participating”, “why am I participating”, and “why you should be participating”. It is these voices that encompass the stereotypes, perceptions, gender norms, the rewards of nursing for men, and men coming to nursing through others, that inform men’s place in nursing and their decision making concerning entering the profession, which are explored further here. 

Qualitative and quantitative data corroborate that men identify a central limitation in considering nursing as a profession revolved around questions of what constitutes a nurse. The feminization of the role, in both professional clinical practice and education domains of nursing, posed a perceived barrier to male participation in nursing [25,35,51]. The pervasive stereotype that male nurses are in some way effeminate and therefore less masculine was a consistent burden to the consideration of nursing as a profession [35,41,51]. While for males already engaged in nursing, they reported feeling a tension between masculinity and the expectations of nursing that required them to adopt a soft masculinity to prevent them from standing out [47], or as something different within clinical practice environments [25,37]. 

While the literature was replete with examples of normalized cultural practices that operate to keep from engaging with nursing as a career, there was also a body of literature that outlines aspects of a career in nursing that was attractive to males [4,23,34,38,45,47]. Employment security was the most often cited justification for males choosing nursing as a career [20,47], while financial remuneration was both attractive and a central reason for males leaving the profession. While intrinsic reward was identified as a reason for choosing nursing as a career, it appeared to come with a caveat. Males recognized that while nursing provided avenues for capitalizing on traits of altruism and caregiving, there was an on-going need to carefully traverse what they perceived to be the precarious line between being a male nurse and the broader societal feminization of caring. The influence of stereotypical ideals that permeate society’s perception of what constitutes a nurse is, again, palpable in the voices of men and their decisions about nursing.

Interestingly, males in the studies reviewed appeared to find nursing a more appealing career option when they were able to identify a role-model who could provide some understanding of what it is that nurses actually do [4,25,26,45,51] and in so doing help to breakdown the stereotypes that surround males in nursing [23]. The time required for males to “arrive” at nursing as a career possibility—often by way of a third party—means that that men are often older when they commence in the profession. Here again one could suggest that the influence of nursing stereotypes operates to keep males at arm’s length without entrée provided by a close insider who can help navigate the fine line between nursing and male identity. 

## 5. Conclusions

While males make up close to 50% of the global population, they represent only 10% of the nursing workforce globally. Nursing is predicted to experience a global shortage in 2025 and finding ever better ways of engaging males in a career in nursing would seem more important now than ever before. Even though there is research suggesting that males who engage in nursing are satisfied with their roles, with a ratio of only 1 in every 10 men contemplating nursing, we have some work to do. This systematic review mixed research synthesis examined the psychological constructs that influence males’ perceptions of nursing, in order to identify those aspects that determine a male’s consideration of nursing as a career.

Arguably, at the core of the issues raised through the previous research reviewed here is a pressing need to re-write what it means to be a nurse, in an effort to dissolve the gendered stereotypes that permeate societies the world over. If nothing else, this review has highlighted that the issues that confound the story of males in nursing are complex and, much like the funambulist’s tightrope walking, they are much more complex that first appreciated. We certainly have an issue as a civilized society when a male is discouraged from showing caring traits out of fear for loosing aspects of one’s masculinity. While we recognize that some of these issues do not encompass nursing alone, but embody contemporary ideas of masculinity, this review has shown us that the long term feminization of nursing means that men in nursing are at all times “different”. They remain “different”, which can be best symbolized through language that is ubiquitous in society. A female nurse is always referred to as “a nurse”, while a male engaged in nursing is always referred to as a “male nurse”. Here the powerful gendered stereotypes in our everyday nomenclature operate to situate one nurse as being somehow different or abnormal in relation to another on the basis of nothing more than gender, and does little to be inclusive of males in nursing. 

## Figures and Tables

**Table 1:** Methodological quality assessment of qualitative articles using the Critical Appraisal Skills Program (CASP) checklist.

Author	A	B	C	D	E	F	G	H	I	J	Total	Quality
Abushaikha et al. [19]	1	1	0.5	0.5	0.5	1	1	0.5	1	1	8.0	Moderate
Christensen et al. [20]	1	1	1	1	1	0	1	1	1	1	9.0	High
Ellis et al. [21]	1	0.5	0.5	0.5	0.5	0	0.5	0.5	1	1	5.5	Exclude
Evans and Frank [22]	0.5	0.5	0.5	0	0.5	0	0	0	0	0.5	2.5	Exclude
Harding [23]	1	1	0.5	1	1	0.5	1	1	0.5	1	8.5	Moderate
Ierardi et al. [24]	1	0.5	0.5	1	1	0	1	0	1	1	7.0	Low
Jamieson et al. [25]	1	0.5	1	0.5	1	1	1	1	1	1	9.0	High
Juliff et al. [26]	1	1	1	0.5	0	1	1	1	1	1	8.5	Moderate
Ndou and Moloko-Phiri [27]	0.5	0.5	0.5	0.5	1	0	1	0.5	0.5	0.5	5.5	Exclude
Meadus and Twomey [4]	0.5	1	1	1	1	1	1	1	1	1	9.5	High
Weaver et al. [28]	1	0.5	1	1	1	0.5	1	1	0.5	1	8.5	Moderate
Whittock and Leonard [29]	1	1	0.5	1	1	0	0	0	0.5	1	6.0	Low

Quality criteria: A: Was there a clear statement of the aims of the research?; B: Is a qualitative methodology appropriate?; C: Was the research design appropriate to address the aims of the research?; D: Was the recruitment strategy appropriate to the aims of the research?; E: Were the data collected in a way that addressed the research issue?; F: Has the relationship between researcher and participants been adequately considered?; G: Have ethical issues been taken into consideration?; H: Was the data analysis sufficiently rigorous? I: Is there a clear statement of findings?; J: How valuable is the research?. Recommendations; 1: yes, 0.5: unsure, 0: no. High-quality paper: scores 9–10; moderate-quality paper: scores 7.5–9; low-quality paper: less than 7.5; exclude: less than 6.

**Table 2:** Methodological quality assessment of quantitative articles using Best Evidence Medical Education (BEME) indicators.

Author	A	B	C	D	E	F	G	H	I	J	K	Total	Quality
Ashkenazi et al. [34]	+	+	+	+	u	+	+	+	+	u	+	9	High
Bartfay et al. [35]	+	+	+	u	u	+	+	+	+	-	+	8	High
Clow et al. [36]	+	+	+	-	-	-	+	+	+	-	+	7	High
Clow et al. [37]	+	+	+	u	-	u	+	+	+	-	+	7	High
Hoffart et al. [38]	-	+	+	-	+	+	+	+	+	u	+	8	High
Loughrey [39]	-	+	+	-	+	+	+	+	+	+	+	9	High
McKenna et al. [40]	-	+	u	+	u	u	+	u	+	u	+	5	Exclude
McLaughlin et al. [41]	u	+	+	+	+	-	+	+	+	+	+	9	High
Meadus and Twomey [42]	-	+	+	+	+	-	u	+	+	u	+	7	High
Rajapaksa and Rothstein [43]	+	+	+	+	u	+	+	+	-	-	+	8	High
Rochlen et al. [44]	+	+	+	u	+	+	+	+	+	u	+	9	High
Stanley et al. [45]	+	+	+	-	+	+	+	+	+	-	+	9	High
Thompson et al. [46]	+	+	+	u	u	u	+	+	+	+	+	8	High
Twomey and Meadus [47]	u	+	+	-	+	u	+	u	+	+	+	7	High

Quality criteria: A: Is the research question(s) or hypothesis clearly stated?; B: Is the subject group appropriate for the study being carried out (number, characteristics, selection, and homogeneity)?; C: Are the methods used (qualitative or quantitative) reliable and valid for the research question and context?; D: Have subjects dropped out? Is the attrition rate less than 50%? For questionnaire-based studies, is the response rate acceptable (60% or above)?; E: Have multiple factors/variables been removed or accounted for where possible?; F: Are the statistical or other methods of results analysis used appropriate?; G: Is it clear that the data justify the conclusions drawn?; H: Could the study be repeated by other researchers?; I: Does the study look forward in time (prospective) rather than backwards (retrospective)?; J: Were all relevant ethical issues addressed?; K: Were results supported by data from more than one source? +: Criterion met; −: Criterion not met; n/a: not applicable [31].

**Table 3:** Features of reviewed studies.

Author	Country	Design	Sample (n)	Population	Data Collection	Data Analysis
Abushaikha et al. [19]	Jordan	Qualitative exploratory	20	Undergraduate nursing, males	Interviews	Inductive content analysis
Ashkenazi et al. [34]	Israel	Not explicit	290	Undergraduate nursing, both sex	Questionnaires	Inferential statistics
Bartfay et al. [35]	Canada	Comparative study	149	Undergraduate nursing and non-nursing, both sex	Questionnaire	Descriptive statistical analysis
Christensen et al. [20]	Australia	Qualitative exploratory	8	Undergraduate nursing, males	Interviews	Descriptive Phenomenological approach
Clow et al. [35]	Canada	Not explicit	145	Undergraduate nursing and non-nursing, both sex	Questionnaires	Inferential statistical analysis
Clow et al. [36]	Canada	Not explicit	164	Undergraduate Psychology, both sex	Questionnaires	Inferential statistical analysis
Harding [23]	Australia	Qualitative exploratory	18	Undergraduate, Postgraduate nursing, males	Interviews	Thematic analysis
Hoffart et al. [38]	USA	Not explicit	3506	Undergraduate nursing, both sex	Questionnaires	Inferential statistical analysis
Ierardi et al. [24]	USA	Qualitative exploratory	7	Associate degree, nursing, males	Interviews	Unclear
Jamieson et al. [25]	New Zealand	Qualitative exploratory	8	Postgraduate nursing, males	Interviews	Thematic analysis
Juliff et al. [26]	Australia	Qualitative exploratory	9	New graduated Registered Nurses, males	Interviews	Interpretative phenomenological analysis
Loughrey [39]	Ireland	Non-experimental descriptive	104	Registered Nurses, males	Questionnaires	Inferential statistical analysis
McLaughlin et al. [41]	Ireland	Longitudinal study, not explicit	384	Undergraduate nursing and non-nursing, both sex	Questionnaires	Inferential statistical analysis
Meadus and Twomey [42]	Canada	Descriptive design	62	Registered Nurses, males	Questionnaires	Descriptive statistical analysis
Meadus and Twomey [3]	Canada	Qualitative exploratory	27	Undergraduate nursing, males	Focus groups	Descriptive Phenomenological approach
Rajapaksa and Rothstein [43]	USA	Not explicit	1589	Former Registered Nurses, both sexes	Secondary Analysis	Inferential statistical analysis
Rochlen et al. [44]	USA	Not explicit	174	Registered Nurses, males	Questionnaires	Inferential statistical analysis
Roth and Coleman [51]	USA	Literature Review	n/a	n/a	Literature Review	Not explicit
Sasa [52]	USA	Concept analysis	n/a	n/a	Concept analysis	Not explicit
Stanley et al. [45]	Australia	Non-experimental descriptive	1055	Registered nurses, enrolled nurses and midwives, both sexes	Questionnaires	Descriptive and inferential statistical analysis
Thompson et al. [46]	USA	Not explicit	109	Nursing students, both sexes	Questionnaires	Inferential statistical analysis
Twomey and Meadus [47]	Canada	Descriptive design	239	Registered Nurses, males	Questionnaires	Descriptive and inferential statistical analysis
Weaver et al. [28]	Australia	Qualitative	n/a	n/a	Not explicit	Not explicit
Whittock and Leonard [29]	UK	Qualitative exploratory	42	Pre and Post Registration, males	Interviews	Thematic analysis, but not explicit
